# The outbreak of Crimean-Congo hemorrhagic fever in Iraq - Challenges and way forward

**DOI:** 10.1016/j.amsu.2022.104382

**Published:** 2022-08-17

**Authors:** Uzair Jafar, Muhammad Usman, Muhammad Ehsan, Aiman Naveed, Muhammad Ayyan, Huzaifa Ahmad Cheema

**Affiliations:** Department of Community Medicine, King Edward Medical University, Lahore, Pakistan

## 1 Introduction

Crimean-Congo hemorrhagic fever (CCHF) is caused by infection with Narivirus, a member of the Bunyavirus family, which is spread by the Ixodes ticks. Although it is named after its early outbreaks in Crimea (1944) and Congo (1969), it is no longer limited to these countries. As per World Health Organization (WHO), today CCHF is endemic to Africa, the Balkans, the Middle East, and many Asian countries [1]. The global prevalence of CCHFV acute infections in humans is estimated to be 22.5%. The case fatality rate can range anywhere from 10 to 40% [2].

Iraq in particular has become an endemic area for CCHF since the first reported case in 1979. Every year many cases of CCHF are reported by health authorities of Iraq, but the increasing disease burden raised alarms in the first half of 2022. From 1^st^ Jan 2022 to 29 May 2022, Iraq reported 212 cases of CCHF. This is already 6 times in comparison to 2021 when only 33 cases were reported throughout the year. 115 (54%) of these 212 cases are suspected and 97 (46%) are laboratory confirmed. There have been 27 deaths related to CCHF this year so far, out of which 14 were suspected and 13 were laboratory-confirmed cases [3]. Almost half of these cases were reported in the Thigar governate (n = 47) of southeast Iraq, while the rest have occurred in 12 different governorates [3].

Livestock animals are often infested with ticks; this puts related people at risk. The lack of adequate livestock spraying during the last two years due to COVID-19 is considered one of the reasons for the outbreak [4]. It is especially concerning in a country like Iraq where cattle farming is very common. Many people who died were either shepherds or butchers or had contact with them.

## 2 Challenges

Inadequate coordination between animal and human sectors regarding disease control is the main challenge to this endemic. The lack of tick control campaigns further adds to the outbreak. There is also a shortage of laboratory kits for the diagnosis of CCHF, especially at district levels. This can lead to misdiagnosis or delayed treatment causing an increase in case fatalities [5]. Healthcare staff working in the hospitals are also at risk because of contracting disease through an undiagnosed patient without proper precautions [6]. The upcoming Eid-ul-Adha during the month of July can worsen the situation because there is trade and slaughter of cattle and animals on a large scale. There was an increase in the number of cases during the last Eid holidays and the month of Ramadan [3]. With over 2 million COVID-19 cases since the start of the pandemic, healthcare in Iraq is under immense pressure. Additional outbreaks will not only cause loss of lives but also huge financial and mental stress on the people of Iraq [7]. Worryingly, COVID-19 can mimic the nonspecific symptoms of CCHF. Misdiagnosis of CCHF as COVID-19 can prove to be fatal. Hence, the proper exclusion of suspected CCHF cases is necessary [8].

## 3 Efforts

There has been an active response to the outbreak by both the Iraqi Ministry of Health (MOH) and WHO. Field visits by teams were conducted to investigate the cases [3]. Health education and awareness about the control and prevention of the virus are being spread by Arabic posters and literature among close contacts and high-risk areas [3]. Healthcare personnel are being properly trained and informed about this outbreak. Clinical case management training was conducted in hospitals dealing with the virus. To make the coordination among animal health, agriculture, veterinarians, and researchers better, WHO has formed a “One-Health approach” where various sectors such as agricultural, environmental and veterinary services collaborate for long-term control and management of CCHF [5]. Epidemiological investigation teams have been employed in the Thigar province by Iraq MOH to investigate the outbreak by doing house-to-house visits and contact tracing [3]. Vector control teams have sprayed acaricides in the affected areas and veterinary hospitals [3]. WHO has collaborated with relevant authorities to make sure the availability of diagnostic kits and other resources is vital to fight this battle [3].

## 4 Recommendations

Insect repellants containing DEET (N, N-diethyl-m-toluamide) should be used by people in high-risk areas like livestock and Agricultural workers. Using proper gloves to handle the animals should be encouraged. The animals showing any signs of infection should be isolated and minimal contact should be established with them [9]. Healthcare workers, in particular, should be well-informed about this occupational hazard and they should use proper precautions while dealing with patients [9]. To minimize the spread of disease on the coming Eid, adequate preventive measures should be enforced while dealing with and slaughtering the animals. Infected animals should be properly screened and treated with pesticide sprays before entering the slaughterhouse [3]. Further research should be done regarding the safety and efficacy of the inactivated vaccine for CCHF currently being tested in Eastern Europe. Ribavirin and other potential treatment options should also be explored and worked upon [9]. WHO and the World Organization for Animal Health should come forward in this difficult time and provide Iraq with all the necessary resources to fight this outbreak. A global partnership for the surveillance and management of CCHF should be formed especially in endemic areas.

## 5 Conclusion

The increasing number of cases of this fatal disease demands an effective controlling strategy. It should be ensured that the appropriate steps for disease prevention and treatment are adopted by the public and the government. The role of the WHO for public awareness and disease surveillance seems to be beneficial for the purpose of disease elimination. Moreover, the healthcare system can facilitate in this regard by developing effective vaccines and treatments for CCHF.

## Ethical approval

N/A.

## Sources of funding

None.

## Author contribution

UJ presented the idea. UJ, MU, and ME conceptualized the idea. UJ, ME, AN, MA and HAC wrote the commentary. All the authors did critical revisions in the manuscript.

## Trail registry number

1. Name of the registry: N/A
2. Unique Identifying number or registration ID: N/A
3. Hyperlink to your specific registration (must be publicly accessible and will be checked): N/A

## Guarantor

N/A.

## Consent

N/A.

## Declaration of competing interest

None declared.
